# Histone acetylation as a new mechanism for bilirubin-induced encephalopathy in the Gunn rat

**DOI:** 10.1038/s41598-018-32106-w

**Published:** 2018-09-12

**Authors:** Eleonora Vianello, Stefania Zampieri, Thomas Marcuzzo, Fabio Tordini, Cristina Bottin, Andrea Dardis, Fabrizio Zanconati, Claudio Tiribelli, Silvia Gazzin

**Affiliations:** 1 Fondazione Italiana Fegato-Onlus, Bldg. Q, AREA Science Park, ss14, Km 163.5, Basovizza, 34149 Trieste Italy; 2 University Hospital Santa Maria della Misericordia, Udine. P.le Santa Maria della Misericordia 15, 33100 Udine, Italy; 3 Department of Medical Sciences, Ospedale di Cattinara, Università degli Studi di Trieste, Strada di Fiume 447, 34149 Trieste, Italy; 4 Cancer Genomics Laboratory, Fondazione Edo ed Elvo Tempia Valenta, Via Malta 3, 13900 Biella, Italy; 5 Computer Science Department, University of Torino, 10100 Torino, Italy

## Abstract

Bilirubin neurotoxicity has been studied for decades and has been shown to affect various mechanisms *via* significant modulation of gene expression. This suggests that vital regulatory mechanisms of gene expression, such as epigenetic mechanisms, could play a role in bilirubin neurotoxicity. Histone acetylation has recently received attention in the CNS due to its role in gene modulation for numerous biological processes, such as synaptic plasticity, learning, memory, development and differentiation. Aberrant epigenetic regulation of gene expression in psychiatric and neurodegenerative disorders has also been described. In this work, we followed the levels of histone 3 lysine 14 acetylation (H3K14Ac) in the cerebellum (Cll) of the developing (2, 9, 17 days after the birth) and adult Gunn rat, the natural model for neonatal hyperbilirubinemia and kernicterus. We observed an age-specific alteration of the H3K14Ac in the hyperbilirubinemic animals. The GeneOntology analysis of the H3K14Ac linked chromatin revealed that almost 45% of H3K14Ac ChiP-Seq TSS-promoter genes were involved in CNS development including maturation and differentiation, morphogenesis, dendritogenesis, and migration. These data suggest that the hallmark Cll hypoplasia in the Gunn rat occurs also *via* epigenetically controlled mechanisms during the maturation of this brain structure, unraveling a novel aspect of the bilirubin-induced neurotoxicity.

## Introduction

Bilirubin toxicity to the CNS has been extensively studied for decades and has been shown to be linked to the activation of multiple complex signal cascades, and affects potential toxic/adaptation mechanisms in the brain through gene expression modulation. Examples include oxidative stress and the antioxidant response, excitotoxicity, inflammation, intracellular trafficking, protein degradation, apoptosis, as well as bilirubin transport and bilirubin oxidization (reviewed in^1^).

Epigenetic processes, such as histone acetylation and DNA methylation, regulate the expression of genes through modifications of DNA structure and accessibility. These regulatory mechanisms often contribute to the onset and progression of human neurological disorders, and are altered by toxic compounds (e.g.: cocaine, alcohol)^2–8^. Indeed, histone acetylation is considered an integral part of brain development and differentiation, synaptic plasticity, learning, memory, and neuron maintenance and survival^9–12^. Notably, it is reported that temporal changes in gene expression by acetylation/deacetylation of gene promoters induce persistent changes in the cell (e.g. cell fate), changes in the neurological behaviour^8^, as well induction of excitotoxicity, calcium overload, oxidative stress, inflammation and apoptosis^13^, with the last five described mechanisms in hyperbilirubinemic animals and humans. This suggests the possibility of a link between the hyperbilirubinemic phenotype and the epigenetic. On this basis, we decided to investigate the effect of hyperbilirubinemia on the epigenetic control of the Cll hypoplasia.

## Results

### Serum bilirubin and cerebellar development

**Figure 1:**
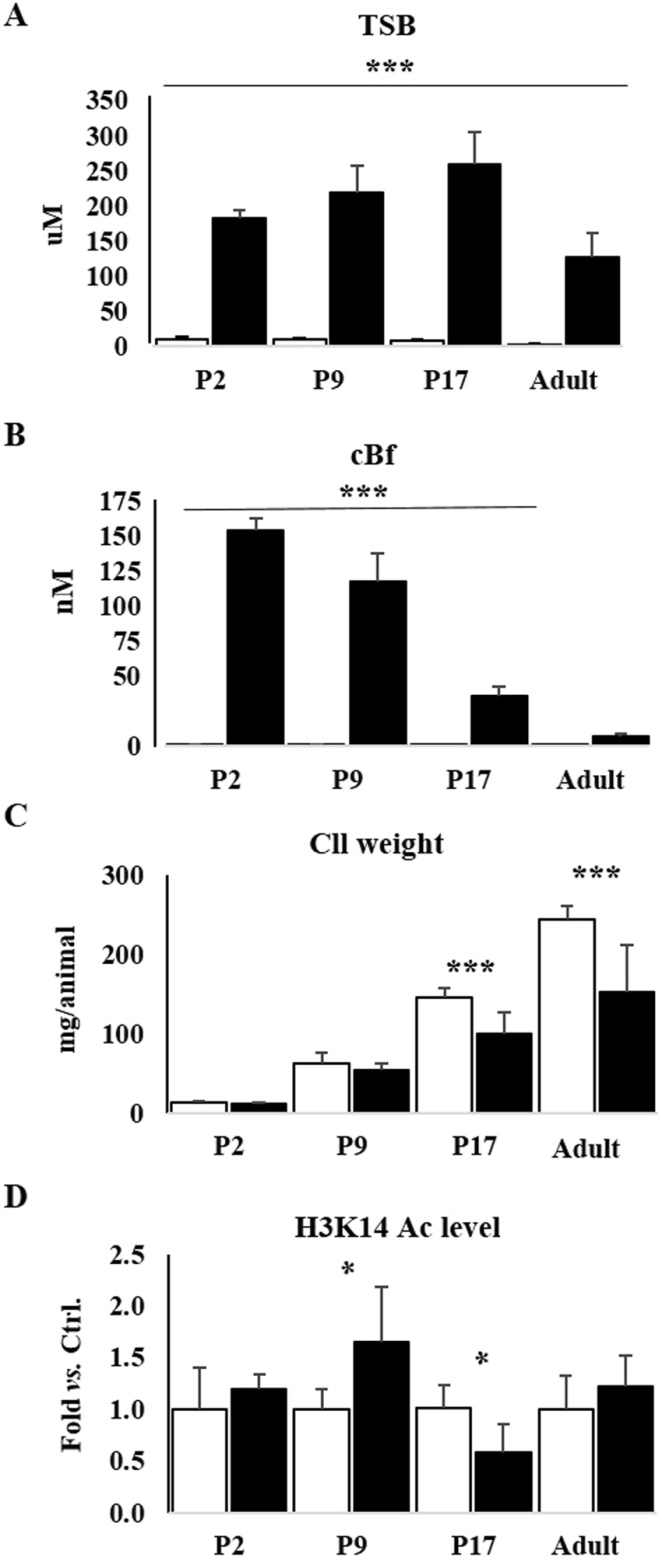
Total Serum Bilirubin (TSB), calculated free bilirubin (cBf) in the blood, cerebellar weight, and Western blot analysis of the level of histone 3 acetylation (H3K14Ac) P: post-natal age in days, Adult: more than 1-year-old. Black bars jj rats, White bars: ctrls. (**A**) TSB (µM); (**B**) cBf (nM), (**C**) Cll weight (mg/animal). Results are expressed as mean ± S.D. of 6–15 animals each group and post-natal age. One way ANOVA followed by Tukey post-test: ***p < 0.001. (**D**) H3K14Ac levels in the Cll of jj animals *vs*. ctrl. Results are as mean ± S.D. of 3–6 animals each group and post-natal age. Unpaired t-test with Welch correction, *p < 0.05 vs. age matched ctrl.

To evaluate the possible correlation between serum bilirubin and the levels of H3K14Ac, we quantified total and free bilirubin in the blood, and the Cll weight in hyperbilirubinemic pups (jj) and normobilirubinemic littermates (control: ctrl) from 2 days after birth (P2) until the adult age. At every post-natal age, the total serum bilirubin (TSB, Fig. 1A) was statistically higher in jj animals compared to ctrl (Σ8.5 lifelong, one-way ANOVA: p ≤ 0.0001, followed by Tukey post-test, p ≤ 0.001). At P2, the TSB was about of 190 µM, peaking at P17 (Σ256 µM), and stabilizing in the adulthood (Σ126 µM), (ever significantly higher than in ctrl, one-way ANOVA: p ≤ 0.0001, followed by Tukey post-test, p ≤ 0.001).

Free bilirubin is the moiety able to cross the blood-brain interfaces leading to neurological damage. In the absence of a reliable method for a direct quantification in the rat, free bilirubin was calculated as previously described^14^. Differently from TSB, the calculated Bf (cBf, Fig. 1B) level in jj pups dropped during development (P2 Σ150 nM, P9 Σ120 nM, P17 Σ35 nM, ever significantly higher than in ctrl, one-way ANOVA: p ≤ 0.0001, followed by Tukey post-test, p ≤ 0.001), felling to the levels not statistically different from those in ctrl in the adult age (adult jj Σ7 nM; One way ANOVA, followed by Tukey post-test, p > 0.05).

Cll weight (Fig. 1C) was similar in jj and ctrl littermates up to P9, becoming significantly different at P17 (Σ30% weight loss *vs*. age-matched ctrl, one way ANOVA followed by Tukey post-test: p < 0.001), and increasing later on (Adult: Σ40%, one way ANOVA followed by Tukey post-test: p < 0.001).

### Western blot analysis of global acetylation of histone H3K14

The follow the level of H3K14Ac in the developing cerebellum of jj and controls rats by Western blot, we used the 07-353 anti-H3K13Ac antibody. At P2, no significant difference was observed in the level of H3K14Ac in the Cll of jj animals compared to age-matched ctrl (Fig. 1D) (unpaired t-test with Welch correction, p = 0.2687). The level of H3K14Ac in jj was significantly increased (1.65 ± 0.54 fold, unpaired t-test with Welch correction, p < 0.0222) at P9 and significantly decreased at P17 (0.67 ± 0.18 fold, unpaired t-test with Welch correction, p < 0.0187). In the adults there was no difference in the level of H3K14Ac between jj and ctrl (unpaired t-test with Welch correction, p = 0.4508).

### ChIP-Seq analysis

**Figure 2:**
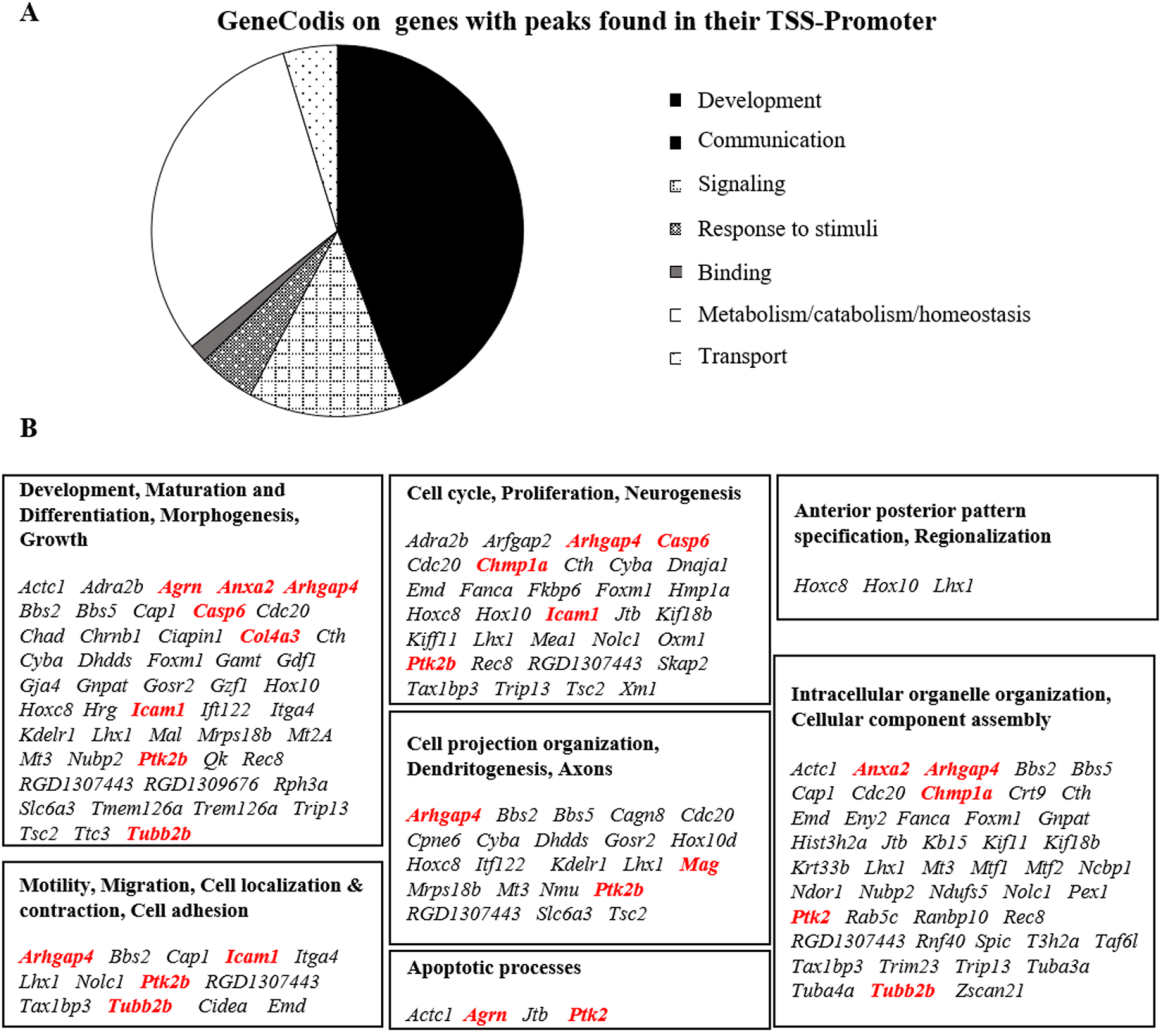
Biological function of the identified Chip-Seq chromatin sequences (**A**) GeneCodis analysis (on genes with peaks found in their TSS-promoter regions) for enriched biological functions. (**B**) List of the 94 (45% of the total found) genes enriched for functions related to the CNS development. In red, genes confirmed by RTqPCR. Hypergeometric p-value ever <0.00005, Corrected (FDR) Hypergeometric p-value < 0.05.

To link the effect of hyperbilirubinemia on H3K14Ac with the genes controlled by this epigenetic mechanism, the 07–353 anti-H3K13Ac antibody used for Western blot analysis was also used to perform chromatin immunoprecipitation, followed by DNA sequencing (ChIP-Seq – full result available on GEO repository # GSE109145). After removal of duplicate DNA fragments and DNA fragments present in both jj and ctrl (physiological genes), 1884 unique DNA sequences were identified. Since variations in the level of histone acetylation in the promoter region positively correlate with gene transcription^9,15^, we focused on peaks identified by ChIP-Seq on the promoter regions (Table 1: 255 genes). As shown in Fig. 2, the functional annotation analysis of the corresponding genes^16–18^ revealed an enrichment for genes involved in CNS development (Σ45%), metabolism & homeostasis (Σ31%), signalling (Σ13%), response to stimuli & communication (Σ5%), transport (Σ5%), and binding (Σ2%).

**Table 1:** Full list of ChIP-Seq TSS-Promoter genes.

Gene Name	Gene Description	Nearest Refseq	Gene Type
*Abcc10*	ATP-binding cassette, subfamily C (CFTR/MRP), member 10	NM_001108201	protein-coding
*Acot13*	acyl-CoA thioesterase 13	NM_001106111	protein-coding
*Acp1*	acid phosphatase 1, soluble	NM_021262	protein-coding
*Acpt*	acid phosphatase, testicular	NM_001107510	protein-coding
*Actc1*	actin, alpha, cardiac muscle 1	NM_019183	protein-coding
*Adra2b*	adrenoceptor alpha 2B	NM_138505	protein-coding
*Agrn*	agrin	NM_175754	protein-coding
*Ahrr*	aryl-hydrocarbon receptor repressor	NM_001024285	protein-coding
*Aldh3a2*	aldehyde dehydrogenase 3 family, member A2	NM_031731	protein-coding
*Alg11*	ALG11, alpha-1,2-mannosyltransferase	NM_001108401	protein-coding
*Alg8*	ALG8, alpha-1,3-glucosyltransferase	NM_001034127	protein-coding
*Amdhd1*	amidohydrolase domain containing 1	NM_001191781	protein-coding
*Anxa2*	annexin A2	NM_019905	protein-coding
*Arfgap2*	ADP-ribosylation factor GTPase activating protein 2	NM_001033707	protein-coding
*Arhgap4*	Rho GTPase activating protein 4	NM_144740	protein-coding
*Asl*	argininosuccinate lyase	NM_021577	protein-coding
Atp6v0e1	ATPase, H+ transporting, lysosomal, V0 subunit e1	NM_053578	protein-coding
Atraid	all-trans retinoic acid-induced differentiation factor	NM_001127526	protein-coding
B3galt4	UDP-Gal:betaGlcNAc beta 1,3-galactosyltransferase, polypeptide 4	NM_133553	protein-coding
Bbs2	Bardet-Biedl syndrome 2	NM_053618	protein-coding
Bbs5	Bardet-Biedl syndrome 5	NM_001108583	protein-coding
Bin2	bridging integrator 2	NM_001012223	protein-coding
Bphl	biphenyl hydrolase-like (serine hydrolase)	NM_001037206	protein-coding
Brd9	bromodomain containing 9	NM_001107453	protein-coding
Cacng8	calcium channel, voltage-dependent, gamma subunit 8	NM_080696	protein-coding
Cap1	CAP, adenylate cyclase-associated protein 1 (yeast)	NM_022383	protein-coding
Casp6	caspase 6	NM_031775	protein-coding
Cblc	Cbl proto-oncogene C, E3 ubiquitin protein ligase	NM_001034920	protein-coding
Cct6a	chaperonin containing Tcp1, subunit 6 A (zeta 1)	NM_001033684	protein-coding
Cdc20	cell division cycle 20	NM_171993	protein-coding
Cers1	ceramide synthase 1	NM_001044230	protein-coding
Chad	chondroadherin	NM_019164	protein-coding
Chmp1a	charged multivesicular body protein 1 A	NM_001083313	protein-coding
Chrnb1	cholinergic receptor, nicotinic, beta 1 (muscle)	NM_012528	protein-coding
Ciapin1	cytokine induced apoptosis inhibitor 1	NM_001007689	protein-coding
Cidea	cell death-inducing DFFA-like effector a	NM_001170467	protein-coding
Clpsl2	colipase-like 2	NM_001135002	protein-coding
Cnksr1	connector enhancer of kinase suppressor of Ras 1	NM_001039011	protein-coding
Col4a3	collagen, type IV, alpha 3	NM_001135759	protein-coding
Col7a1	collagen, type VII, alpha 1	NM_001106858	protein-coding
Cpne6	copine VI (neuronal)	NM_001191113	protein-coding
Cpsf3l	cleavage and polyadenylation specific factor 3-like	NM_001033892	protein-coding
Cpsf4	cleavage and polyadenylation specific factor 4	NM_001012351	protein-coding
Crcp	CGRP receptor component	NM_053670	protein-coding
Cth	cystathionine gamma-lyase	NM_017074	protein-coding
Ctr9	CTR9 homolog, Paf1/RNA polymerase II complex component	NM_001100661	protein-coding
Cyb5r1	cytochrome b5 reductase 1	NM_001013126	protein-coding
Cyba	cytochrome b-245, alpha polypeptide	NM_024160	protein-coding
Ddb1	damage-specific DNA binding protein 1, 127 kDa	NM_171995	protein-coding
Ddb2	damage specific DNA binding protein 2	NM_001271346	protein-coding
Ddias	DNA damage-induced apoptosis suppressor	NM_001126294	protein-coding
Ddit4l2	DNA-damage-inducible transcript 4-like 2	NM_080399	protein-coding
Ddx55	DEAD (Asp-Glu-Ala-Asp) box polypeptide 55	NM_001271326	protein-coding
Ddx56	DEAD (Asp-Glu-Ala-Asp) box helicase 56	NM_001004211	protein-coding
Dhdds	dehydrodolichyl diphosphate synthase subunit	NM_001011978	protein-coding
Dmrtc2	DMRT-like family C2	NM_001109140	protein-coding
Dnaja1	DnaJ (Hsp40) homolog, subfamily A, member 1	NM_022934	protein-coding
Eif3e	eukaryotic translation initiation factor 3, subunit E	NM_001011990	protein-coding
Emc3	ER membrane protein complex subunit 3	NM_001008355	protein-coding
Emd	emerin	NM_012948	protein-coding
Entpd6	ectonucleoside triphosphate diphosphohydrolase 6	NM_053498	protein-coding
Eny2	enhancer of yellow 2 homolog (Drosophila)	NM_001130580	protein-coding
Ephx2	epoxide hydrolase 2, cytoplasmic	NM_022936	protein-coding
Fam151a	family with sequence similarity 151, member A	NM_001005558	protein-coding
Fam178b	family with sequence similarity 178, member B	NM_001122653	protein-coding
Fam192a	family with sequence similarity 192, member A	NM_001014014	protein-coding
Fanca	Fanconi anemia, complementation group A	NM_001108455	protein-coding
Fbxo44	F-box protein 44	NM_001191576	protein-coding
Fdxr	ferredoxin reductase	NM_024153	protein-coding
Fgfr1op2	FGFR1 oncogene partner 2	NM_201421	protein-coding
Fkbp6	FK506 binding protein 6	NM_001105922	protein-coding
Foxm1	forkhead box M1	NM_031633	protein-coding
Fyco1	FYVE and coiled-coil domain containing 1	NM_001106870	protein-coding
Gamt	guanidinoacetate N-methyltransferase	NM_012793	protein-coding
Gdf1	growth differentiation factor 1	NM_001044240	protein-coding
Gja4	gap junction protein, alpha 4	NM_021654	protein-coding
Gjd4	gap junction protein, delta 4	NM_001100487	protein-coding
Gna15	guanine nucleotide binding protein, alpha 15	NM_053542	protein-coding
Gng5	guanine nucleotide binding protein (G protein), gamma 5	NM_024377	protein-coding
Gnmt	glycine N-methyltransferase	NM_017084	protein-coding
Gnpat	glyceronephosphate O-acyltransferase	NM_053410	protein-coding
Gosr2	golgi SNAP receptor complex member 2	NM_031685	protein-coding
Gpalpp1	GPALPP motifs containing 1	NM_001024875	protein-coding
Gtf2e1	general transcription factor IIE, polypeptide 1, alpha	NM_001100556	protein-coding
Gtsf1	gametocyte specific factor 1	NM_001079707	protein-coding
Gzf1	GDNF-inducible zinc finger protein 1	NM_001107788	protein-coding
Hcfc1r1	host cell factor C1 regulator 1 (XPO1-dependent)	NM_001100492	protein-coding
Higd2a	HIG1 hypoxia inducible domain family, member 2 A	NM_001106102	protein-coding
Hist3h2a	histone cluster 3, H2a	NM_021840	protein-coding
Hist3h2bb	histone cluster 3, H2bb	NM_001109641	protein-coding
Hoxc8	homeobox C8	NM_001177326	protein-coding
Hoxd10	homeo box D10	NM_001107094	protein-coding
Hrg	histidine-rich glycoprotein	NM_133428	protein-coding
Icam1	intercellular adhesion molecule 1	NM_012967	protein-coding
Idua	iduronidase, alpha-L-	NM_001172084	protein-coding
Ift122	intraflagellar transport 122	NM_001009416	protein-coding
Ikzf5	IKAROS family zinc finger 5	NM_001107555	protein-coding
Il17rb	interleukin 17 receptor B	NM_001107290	protein-coding
Itga4	integrin, alpha 4	NM_001107737	protein-coding
Jagn1	jagunal homolog 1	NM_001044272	protein-coding
Jtb	jumping translocation breakpoint	NM_019213	protein-coding
Kb15	type II keratin Kb15	NM_001008825	protein-coding
Kcne5	potassium channel, voltage-gated Isk-related subfamily E regulatory beta subunit 5	NM_001101003	protein-coding
Kdelr1	KDEL (Lys-Asp-Glu-Leu) endoplasmic reticulum protein retention receptor 1	NM_001017385	protein-coding
Kiaa0895l	hypothetical protein LOC688736	NM_001044292	protein-coding
Kif11	kinesin family member 11	NM_001169112	protein-coding
Kif18b	kinesin family member 18B	NM_001039019	protein-coding
Klrd1	killer cell lectin-like receptor, subfamily D, member 1	NM_012745	protein-coding
Krt33b	keratin 33B	NM_001008819	protein-coding
Lars2	leucyl-tRNA synthetase 2, mitochondrial	NM_001108787	protein-coding
Leng1	leukocyte receptor cluster (LRC) member 1	NM_001106218	protein-coding
Lhx1	LIM homeobox 1	NM_145880	protein-coding
LOC100912214	uncharacterized LOC100912214	NR_131101	ncRNA
LOC103689982	lysophospholipid acyltransferase 7	NM_001313940	protein-coding
LOC288913	similar to LEYDIG CELL TUMOR 10 KD PROTEIN	NM_198728	protein-coding
LOC498154	hypothetical protein LOC498154	NM_001025033	protein-coding
LOC688925	similar to Glutathione S-transferase alpha-4	NM_001270386	protein-coding
Lrrc14	leucine rich repeat containing 14	NM_001024354	protein-coding
Lrrc27	leucine rich repeat containing 27	NM_001113789	protein-coding
Lrrc36	leucine rich repeat containing 36	NM_001004088	protein-coding
Lrrc51	leucine rich repeat containing 51	NM_001106284	protein-coding
Lypd3	Ly6/Plaur domain containing 3	NM_021759	protein-coding
Lzic	leucine zipper and CTNNBIP1 domain containing	NM_001013241	protein-coding
Lztfl1	leucine zipper transcription factor-like 1	NM_001024266	protein-coding
Maf1	MAF1 homolog, negative regulator of RNA polymerase III	NM_001014085	protein-coding
Mag	myelin-associated glycoprotein	NM_017190	protein-coding
Mal	mal, T-cell differentiation protein	NM_012798	protein-coding
Mboat7	membrane bound O-acyltransferase domain containing 7	NM_001134978	protein-coding
Mcemp1	mast cell-expressed membrane protein 1	NM_001134602	protein-coding
Mea1	male-enhanced antigen 1	NM_001044286	protein-coding
Med11	mediator complex subunit 11	NM_001105799	protein-coding
Mir137	microRNA 137	NR_031883	ncRNA
Mir207	microRNA 207	NR_032107	ncRNA
Mir338	microRNA 338	NR_031783	ncRNA
Mir3562	microRNA 3562	NR_037344	ncRNA
Misp	mitotic spindle positioning	NM_001109284	protein-coding
Mrpl43	mitochondrial ribosomal protein L43	NM_001107598	protein-coding
Mrps18b	mitochondrial ribosomal protein S18B	NM_212534	protein-coding
Mrps25	mitochondrial ribosomal protein S25	NM_001025408	protein-coding
Mt2A	metallothionein 2A	NM_001137564	protein-coding
Mt3	metallothionein 3	NM_053968	protein-coding
Mterf3	mitochondrial transcription termination factor 3	NM_199387	protein-coding
Mtf1	metal-regulatory transcription factor 1	NM_001108677	protein-coding
Mtf2	metal response element binding transcription factor 2	NM_001100898	protein-coding
Myeov2	myeloma overexpressed 2	NM_001109044	protein-coding
Naa38	N(alpha)-acetyltransferase 38, NatC auxiliary subunit	NM_001105794	protein-coding
Ncbp1	nuclear cap binding protein subunit 1	NM_001014785	protein-coding
Ncoa4	nuclear receptor coactivator 4	NM_001034007	protein-coding
Ndor1	NADPH dependent diflavin oxidoreductase 1	NM_001107818	protein-coding
Ndufb8	NADH dehydrogenase (ubiquinone) 1 beta subcomplex 8	NM_001106360	protein-coding
Ndufs5	NADH dehydrogenase (ubiquinone) Fe-S protein 5	NM_001030052	protein-coding
Ndufv3	NADH dehydrogenase (ubiquinone) flavoprotein 3	NM_022607	protein-coding
Nipsnap1	nipsnap homolog 1 (C. elegans)	NM_001100730	protein-coding
Nme3	NME/NM23 nucleoside diphosphate kinase 3	NM_053507	protein-coding
Nmi	N-myc (and STAT) interactor	NM_001034148	protein-coding
Nmu	neuromedin U	NM_022239	protein-coding
Nob1	NIN1/RPN12 binding protein 1 homolog	NM_199086	protein-coding
Nolc1	nucleolar and coiled-body phosphoprotein 1	NM_022869	protein-coding
Nr2c2ap	nuclear receptor 2C2-associated protein	NM_001047104	protein-coding
Nsl1	NSL1, MIS12 kinetochore complex component	NM_001109083	protein-coding
Ntpcr	nucleoside-triphosphatase, cancer-related	NM_001134573	protein-coding
Ntsr1	neurotensin receptor 1	NM_001108967	protein-coding
Nubp2	nucleotide binding protein 2	NM_001011891	protein-coding
Nudt2	nudix (nucleoside diphosphate linked moiety X)-type motif 2	NM_207596	protein-coding
Olr437	olfactory receptor 437	NM_001109347	protein-coding
Olr760	olfactory receptor 760	NM_001001069	protein-coding
Ovca2	ovarian tumor suppressor candidate 2	NM_001109036	protein-coding
Pcdha3	protocadherin alpha 3	NM_053941	protein-coding
Pctp	phosphatidylcholine transfer protein	NM_017225	protein-coding
Pex1	peroxisomal biogenesis factor 1	NM_001109220	protein-coding
Phlda2	pleckstrin homology-like domain, family A, member 2	NM_001100521	protein-coding
Phldb3	pleckstrin homology-like domain, family B, member 3	NM_001191622	protein-coding
Pigp	phosphatidylinositol glycan anchor biosynthesis, class P	NM_001099758	protein-coding
Plcxd2	phosphatidylinositol-specific phospholipase C, X domain containing 2	NM_001134481	protein-coding
Plp2	proteolipid protein 2 (colonic epithelium-enriched)	NM_207601	protein-coding
Pmf1	polyamine-modulated factor 1	NM_001191568	protein-coding
Pnldc1	poly(A)-specific ribonuclease (PARN)-like domain containing 1	NM_001025724	protein-coding
Polr3d	polymerase (RNA) III (DNA directed) polypeptide D	NM_001031653	protein-coding
Pou6f1	POU class 6 homeobox 1	NM_001105746	protein-coding
Ppp1r11	protein phosphatase 1, regulatory (inhibitor) subunit 11	NM_212542	protein-coding
Ppt2	palmitoyl-protein thioesterase 2	NM_019367	protein-coding
Psmg4	proteasome (prosome, macropain) assembly chaperone 4	NM_001109543	protein-coding
Ptcd1	pentatricopeptide repeat domain 1	NM_001109665	protein-coding
Ptk2b	protein tyrosine kinase 2 beta	NM_017318	protein-coding
Qk	quaking	NM_001115021	protein-coding
Rab3gap2	RAB3 GTPase activating protein subunit 2	NM_001008294	protein-coding
Rab5c	RAB5C, member RAS oncogene family	NM_001105840	protein-coding
Rad51ap1	RAD51 associated protein 1	NM_001079711	protein-coding
Ranbp10	RAN binding protein 10	NM_001135875	protein-coding
Rec8	REC8 meiotic recombination protein	NM_001011916	protein-coding
Rfc2	replication factor C (activator 1) 2	NM_053786	protein-coding
RGD1307443	similar to mKIAA0319 protein	NM_001197023	protein-coding
RGD1309188	similar to hypothetical protein BC011833	NM_001108129	protein-coding
RGD1309676	similar to RIKEN cDNA 5730469M10	NM_001014140	protein-coding
RGD1311703	similar to sid2057p	NM_001013898	protein-coding
RGD1359334	similar to hypothetical protein FLJ20519	NM_001007638	protein-coding
RGD1559909	RGD1559909	NM_001108678	protein-coding
RGD1560608	similar to novel protein	NM_001109280	protein-coding
RGD1562683	RGD1562683	NM_001108314	protein-coding
RGD1563714	RGD1563714	NM_001126297	protein-coding
RGD1564036	similar to RIKEN cDNA 3010026O09	NM_001109030	protein-coding
Ribc2	RIB43 A domain with coiled-coils 2	NM_001013949	protein-coding
Rnf40	ring finger protein 40, E3 ubiquitin protein ligase	NM_153471	protein-coding
Rph3a	rabphilin 3A	NM_133518	protein-coding
Rpl27	ribosomal protein L27	NM_022514	protein-coding
Rpl27a	ribosomal protein L27a	NM_001106290	protein-coding
Rspry1	ring finger and SPRY domain containing 1	NM_001100945	protein-coding
Rxfp3	relaxin/insulin-like family peptide receptor 3	NM_001008310	protein-coding
Sart3	squamous cell carcinoma antigen recognized by T-cells 3	NM_001107156	protein-coding
Scly	selenocysteine lyase	NM_001007755	protein-coding
Sert1	Sertoli cell protein 1	NR_130708	ncRNA
Sfxn3	sideroflexin 3	NM_022948	protein-coding
Skap2	src kinase associated phosphoprotein 2	NM_130413	protein-coding
Slc19a2	solute carrier family 19 (thiamine transporter), member 2	NM_001030024	protein-coding
Slc25a54	solute carrier family 25, member 54	NM_001109640	protein-coding
Slc43a3	solute carrier family 43, member 3	NM_001107743	protein-coding
Slc5a6	solute carrier family 5 (sodium/multivitamin and iodide cotransporter), member 6	NM_130746	protein-coding
Slc6a20	solute carrier family 6 (proline IMINO transporter), member 20	NM_133296	protein-coding
Slc6a3	solute carrier family 6 (neurotransmitter transporter), member 3	NM_012694	protein-coding
Snrnp35	small nuclear ribonucleoprotein 35 (U11/U12)	NM_001014127	protein-coding
Snrpb2	small nuclear ribonucleoprotein polypeptide B”	NM_001108592	protein-coding
Spag7	sperm associated antigen 7	NM_001107016	protein-coding
Spata33	spermatogenesis associated 33	NM_001106195	protein-coding
Spata5	spermatogenesis associated 5	NM_001108549	protein-coding
Spic	Spi-C transcription factor (Spi-1/PU.1 related)	NM_001108080	protein-coding
Stam	signal transducing adaptor molecule (SH3 domain and ITAM motif) 1	NM_001109121	protein-coding
Stk19	serine/threonine kinase 19	NM_001013197	protein-coding
Susd3	sushi domain containing 3	NM_001107341	protein-coding
Tada3	transcriptional adaptor 3	NM_001025734	protein-coding
Taf6l	TAF6-like RNA polymerase II, p300/CBP-associated factor (PCAF)-associated factor	NM_001107575	protein-coding
Tax1bp3	Tax1 (human T-cell leukemia virus type I) binding protein 3	NM_001025419	protein-coding
Tbc1d25	TBC1 domain family, member 25	NM_001106955	protein-coding
Tbcb	tubulin folding cofactor B	NM_001040180	protein-coding
Them4	thioesterase superfamily member 4	NM_001025017	protein-coding
Tmem109	transmembrane protein 109	NM_001007736	protein-coding
Tmem126a	transmembrane protein 126 A	NM_001011557	protein-coding
Tnxa-ps1	tenascin XA, pseudogene 1	NR_024118	pseudo
Trappc1	trafficking protein particle complex 1	NM_001039378	protein-coding
Trim23	tripartite motif-containing 23	NM_001100637	protein-coding
Trip13	thyroid hormone receptor interactor 13	NM_001011930	protein-coding
Trip4	thyroid hormone receptor interactor 4	NM_001134981	protein-coding
Trmt112	tRNA methyltransferase 11-2 homolog (S. cerevisiae)	NM_001106330	protein-coding
Tsc2	tuberous sclerosis 2	NM_012680	protein-coding
Tstd2	thiosulfate sulfurtransferase (rhodanese)-like domain containing 2	NM_001108663	protein-coding
Ttc3	tetratricopeptide repeat domain 3	NM_001108315	protein-coding
Tuba3a	tubulin, alpha 3A	NM_001040008	protein-coding
Tuba4a	tubulin, alpha 4A	NM_001007004	protein-coding
Tubb2b	tubulin, beta 2B class IIb	NM_001013886	protein-coding
Ufsp2	UFM1-specific peptidase 2	NM_001014142	protein-coding
Vmp1	vacuole membrane protein 1	NM_138839	protein-coding
Vwa7	von Willebrand factor A domain containing 7	NM_212499	protein-coding
Zbtb26	zinc finger and BTB domain containing 26	NM_001107840	protein-coding
Zfp142	zinc finger protein 142	NM_001108225	protein-coding
Zfp597	zinc finger protein 597	NM_153732	protein-coding
Zscan21	zinc finger and SCAN domain containing 21	NM_001012021	protein-coding

### Morphological features of the Gunn rat Cll

**Figure 3:**
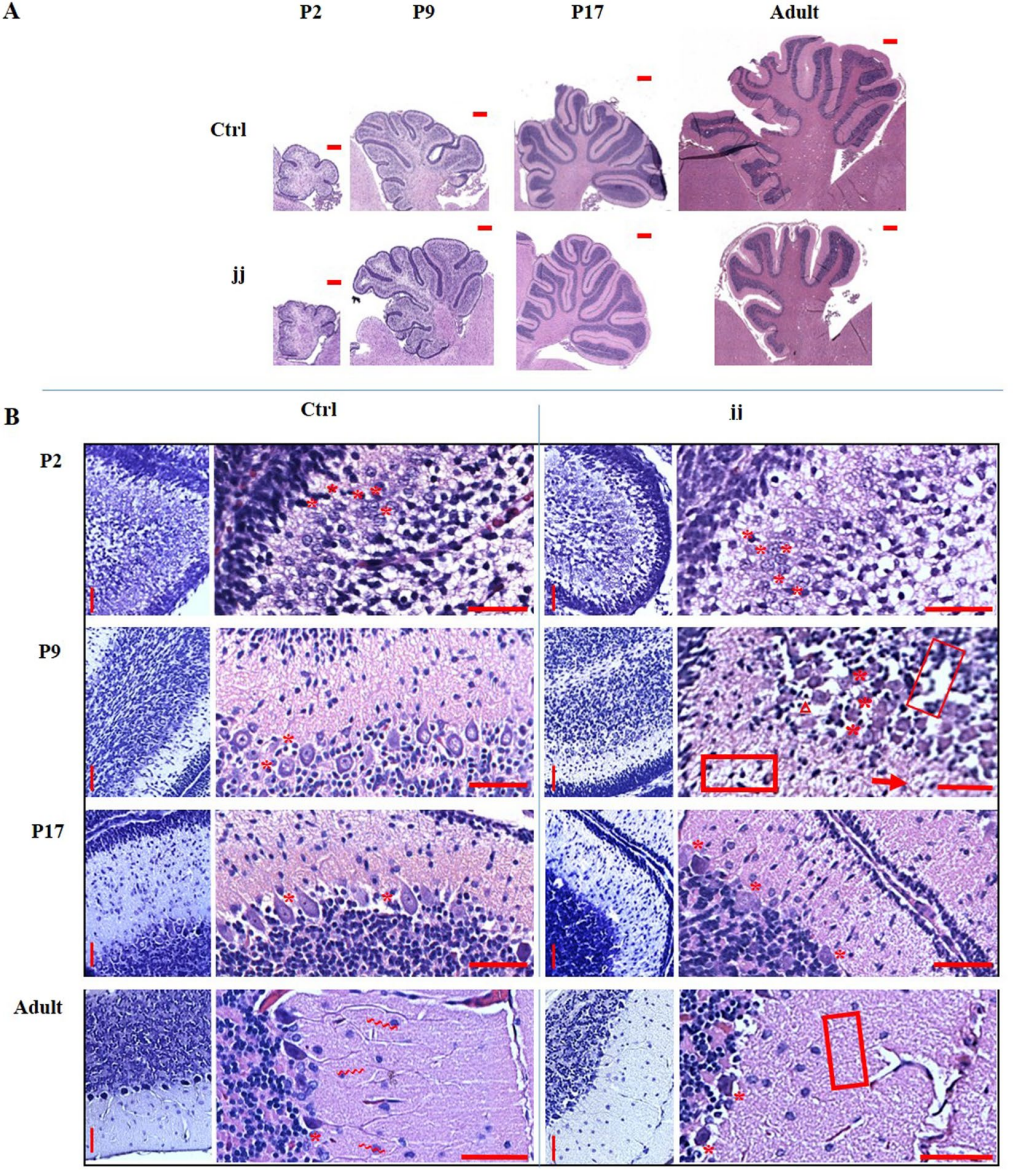
Histological finding (**A**) Full Cll images (scale bar 400 µm) showing the normal development (ctrl, upper series of pictures) and the progression of the Cll hypoplasia in jj animals (lower series of pictures). (**B**) Details (scale bar 100 µm) of the major histological alterations in the developing Cll of jj rat *vs*. age matched ctrl. P: post-natal age in days, Adult: more than 1 year old. *Purkinje cells (PCs); >PC’s neurites; ∆ microgliosis; [] extracellular matrix alteration; → oedema. 2–3 animals each genotype/age have been used. Miniatures: Nissl stain. Larger pictures: Haematoxylin & Eosin.

Since our results strongly suggested an impact of bilirubin on the genetic program of CNS maturation, we systematically followed the histological development of the cerebellum of jj rats in the attempt to interpret the genetic results. No morphological alterations between jj and ctrl were obvious at P2 (Fig. 3A,B). In both jj and ctrl animals, Purkinje cells were organized in 3–5 layers, with a round/oval shape and a reticulated cytoplasm (Fig. 3B). At P9, in spite of a conserved architecture, signs of cellular sufferance/death, microgliosis, extracellular matrix abnormalities and edema were evident in jj pups. PCs in ctrl displayed a clear definition of the plasma-membrane, cytoplasm, and nuclear areas, and a round/drop shape, and were organized in 3/1 layers. On the contrary, in jj pups, PCs were largely present in 4/2 layers, with an undefined, irregular shape. At P17, microgliosis and signs of cellular sufferance were still present in jj rats. PCs in ctrl were well differentiated, with a drop shape, and almost completely organized in a single layer, diffusely in 2/1 layers and still presenting the altered morphology described at P9 in jj. In the adult animal, the effect of Cll hypoplasia was well appreciable, with a less developed structure characterized by large spaces between the folia (Fig. 3A). Microgliosis was reduced but still present. No PC’s neurites were visible in jj rats, where PCs appeared atrophic and apoptotic (Fig. 3B).

### RTqPCR analysis of selected genes

**Figure 4:**
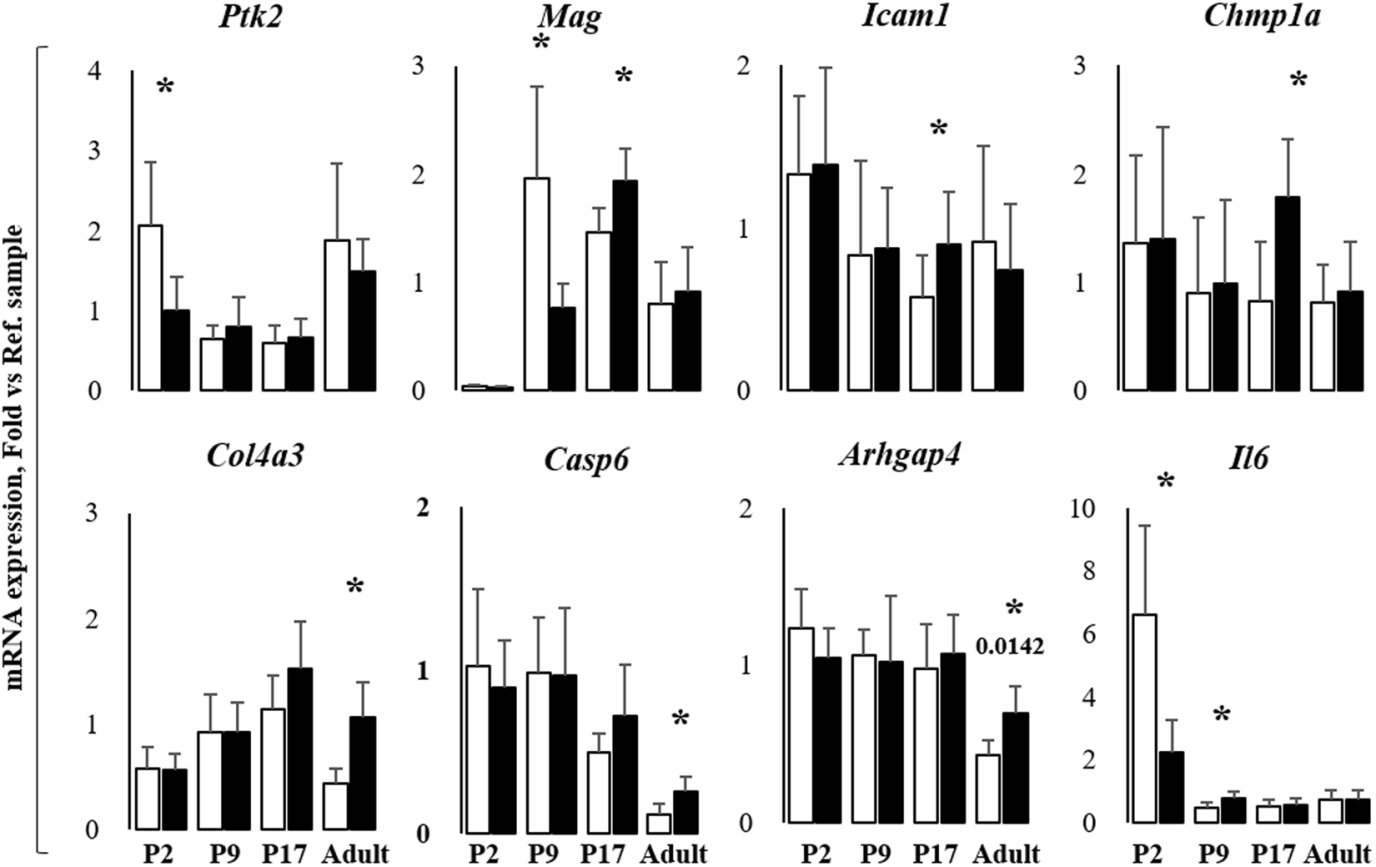
Analysis of the expression of selected genes involved in CNS development *Arghap4*: Rho GTP-ase activating protein 4; *Casp6*: Caspase 6; *Chmp1a*: Charged multi-vesicular body protein 1a; *Col4a3*: Collagenase 4 a3; *Icam1*: Intracellular adhesion molecule 1; *Mag*: Myelin-associated glycoprotein; *Ptk2*: Protein tyrosine kinase 2 beta; *Il6*: Interleukin 6. P: post-natal age in days, Adult: more than 1-year-old. White bars: ctrl; Black bars: jj. Results are expressed as mean ± S.D. of 6 animals each genotype/age. Unpaired t-test with Welch correction, *p < 0.05; **p < 0.05; ***p < 0.005 vs. age-matched controls.

Due to the surprising percentage of enrichment for genes involved in CNS development, we decided to confirm and quantify the epigenetic control of a selected panel of genes, by assessing their expression by RTqPCR (selected genes are those in red in Fig. 2B, in which their biological functions based on the Gene Ontology analysis are indicated. RTqPCR results are in Fig. 4). *Ptk2* (protein tyrosine kinase 2 beta, considered a key gene in neurite outgrowth and elongation, synapses formation, and actin reorganization^19^), was significantly down-regulated in P2 jj pups (Σ2 fold *vs*. age-matched ctrl, unpaired t-test with Welch correction, p < 0.047), normalizing thereafter. *Mag* (myelin-associated glycoprotein), barely detectable immediately after birth, was highly expressed in ctrl and Σ2.5 fold down-regulated in jj pups at P9 (unpaired t-test with Welch correction, p < 0.0402), reversing to a Σ1.2 fold up-regulation at P17 (unpaired t-test with Welch correction, p < 0.0306). *Icam1* (intracellular adhesion molecule 1, expressed mainly by the endothelial cells forming the blood-brain barrier, involved in cell adhesion, leucocytes^20^ and monocytes extravasation^21^, and morphogenesis) was up-regulated 1.6 fold in P17 jj rats (unpaired t-test with Welch correction, p < 0.0416). Similarly, we observed a Σ2.2 fold increase (unpaired t-test with Welch correction, p < 0.0315) of *Chmp1a* (charged multi-vesicular body protein 1a, regulating the neural progenitor cell proliferation^22^). In adult jj Cll, *Col4a3* (collagenase 4a3, the major structural component of the basal membrane, involved in the extracellular matrix remodeling^23^, providing the functional compartmentalization of the brain by clustering of growth factors, neurotransmitters/ions receptors, as well contributing to migration and differentiation^24^), *Casp6* (caspase 6 - proliferation and morphogenesis – Fig. 2B), and *Arghap4* (Rho GTPase-activating protein, inhibiting the cell motility and axon outgrowth *via* regulating the cytoskeleton dynamics^25^) were upregulated Σ2.5fold (unpaired t-test with Welch correction, p < 0.00547), Σ1.9fold (unpaired t-test with Welch correction, p < 0.0287) and Σ1.6 fold (unpaired t-test with Welch correction, p < 0.0142) respectively. No modulation of *Anxa2* (annexin2), *Agrn* (Agrin), and *Tubb2b* (Tubulin2b) was detected at any post-natal age in jj rats (data not shown). *Il6* (intron region segment resulting from ChIP-Seq analysis) was also investigated. In ctrl animals the *Il6* level rapidly decreases from P2 to P9, stabilizing thereafter. In jj pups, a significant down-regulation of *Il6* was present immediately after birth compared to ctrl animals (Σ2.9fold, unpaired t-test with Welch correction, p < 0.0315), while a 1.65 fold up-regulation was noticed at P9 (unpaired t-test with Welch correction, p < 0.0248), normalizing later on.

## Discussion

Cll hypoplasia is a hallmark of hyperbilirubinemia in rodents^26–29^, and cerebellar involvement with morphological and behavioral abnormalities has also been reported in severely hyperbilirubinemic neonates^30–32^. Inflammation and oxidative stress are considered the major mechanisms of bilirubin neurotoxicity, whereas the impact of hyperbilirubinaemia on CNS development has been only marginally envisaged, and evaluated mostly by *in vitro* experiments^33,34^.

Unexpectedly, the known inflammatory or oxidant effectors of bilirubin neurotoxicity have been not identified in our data (ChIP-Seq, followed by Gene Ontology analysis), revealing that 45% of genes displaying a Histone 3 lysine 14 acetylation are related to CNS development. Indeed, only 3 genes among all the 255 identified TSS- Promoter sequences have been previously reported in the literature for their association with hyperbilirubinemia, namely *myelin*^28,31,32,34^, *tubulin*^35^, and *Icam1*^36^.

The down-regulation of *Mag* has been reported in *in vitro* studies, in agreement with the defective myelination observed both in bilirubin neurotoxicity models^28,34^ and neonates^32^. *Mag* down-regulation is also a known consequence of bilirubin-induced perturbation of the oligodendrocytes maturation. A possible additional link between what has been previously described and the present results is the fact that histone acetylation is a known mechanism controlling oligodendrocyte differentiation and myelin production, both in physiological CNS development and in repair processes after demyelination^6,10^.

Our data are in agreement with the literature also in relation to *Il6*, whose intron sequence was identified by ChIP-Seq analysis. Il6 is a well-known effector of bilirubin neurotoxicity and possibly linked with the reported defective myelination. In fact, apart from the possible inflammatory activity, *Il6* is involved in oligodendrogenesis^37,38^, a process active up to P45 in rodents and 2 years in humans^39^, and reactivated in pathological conditions. During reactivation, injured neurons and oligodendrocytes may reactivate myelin synthesis by overexpressing *Il6* and its receptor (*Il6r/CD126*), restoring normal behavior in injured animals^10,40^.

Both *Mag* and *Il6* present a fluctuating behavior, being significantly down-regulated in the early post-natal life, and reverting thereafter to the level of age-matched controls (Fig. 4). Notably, in our work, *IL6* modulation (P9) precedes *Mag* increase (P17), supporting the inductor role of Il6 in myelination described in the literature^10,40^. The fluctuating expression of *Il6* and *Mag* (firstly up-, then down regulated), is present also for H3K14Ac levels, increasing at P9, and reverting under the level of age-matched controls at P17, and normalizing in the adult age.

The regulation of the other genes is more difficult to be analyzed since they are very new in the bilirubin field and no data are provided by literature. While we still have to confirm the role of the various genes identified in this study through methods such as gene silencing *in vitro*, our work suggests that the epigenetic impairment of neurodevelopmental processes in hyperbilirubinemia may be a relevant mechanism of bilirubin neurotoxicity. It is worth mentioning that *Chmp1a, Arghap4*, *Casp6, Ptk2, Col4a3* are genes involved in key steps of brain development as proliferation, migration, morphogenesis, neurite outgrowth and elongation, synaptogenesis, extracellular matrix formation and compartmentalization, as well the pathological axonal degeneration and apoptosis observed^19,22,25,41,42^ in jj rats. By adding epigenetic dysregulation to the list of the mechanisms related to bilirubin-induced neuronal damage, we can confirm and expand the concept of a widespread toxic effect of the pigment on the CNS^43^, improving our understanding of the cellular and molecular mechanisms of bilirubin induced damage to CNS.

## Materials and Methods

### Animals

Gunn rats (Hds Blue:Gunn-UDPGT^j^, P2, 9, 17; P ± 1 day. Adult = more than 1 year old) were obtained from the SPF animal facility of CBM S.c.a.r.l. (AREA Science Park, Basovizza). Ages were selected based on previous evidence^26,44^. Animals were housed in a temperature-controlled environment (22 ± 2 °C), on a 12 hours light/dark schedule, and ad-libitum access to food and water. The study was approved by the animal care and use committee of the CBM Scarl and the competent Italian Ministry. All procedures were performed according to the Italian Law (decree 87-848) and European Community directive (86-606-ECC). Maximal effort to minimize the number of the animals used and their sufferance was done.

### TSB, cBf and Cerebellum weight quantification

Serum and Cll were collected as previously described^26,45^. In brief, blood samples were collected during the sacrifice (decapitation under urethane anaesthesia 1.0–1.2 g/kg IP) and centrifuged at 2000 rpm, 20 min RT. Total serum bilirubin (TSB) was quantified by the diazo reaction, as previously described^26^. Free bilirubin was calculated (cBf) by applying the formula and the albumin-bilirubin dissociation constants for Gunn pups detailed in literature^14^. Cerebellum was dissected immediately after the sacrifice, and the weight recorded by a precision balance.

### Western blot analysis of the levels of H3K14Ac

Western blot was performed as previously described^44,45^. In brief, Cll were mechanically homogenized by glass-glass Dounce (in 0.25 M sucrose, 40.2 mM KH_2_PO_4_, 9.8 mM K_2_HPO_4_, 1 mM EDTA, 0.1 mM DTT, pH 7.4), and total protein concentration quantified by the Bicinchoninic Acid Protein Assay following the supplier instruction (B-9643 and C2284, Sigma, Missouri, USA). 25 μg of Cll whole extract proteins were denatured (10% of β-mercaptoethanol -Sigma Chemical, St. Louis, MO, USA, plus 5 min boiling), separated by 12% SDS-PAGE by electrophoresis in a Hoefer SE 250 System (Amersham BioSciences, UK), and electro-transferred onto immune-blot PVDF membranes (0.2 μm; Whatman Schkleicher and Schuell, Dassel, Germany) at 100 V for 60 min (Bio-Rad Laboratories, Hercules, CA, USA). Efficiency of the transfer was assessed by lack of Coomassie blue coloration of the gel after blotting, and Ponceau staining of the PVDF membrane (both chemicals: Sigma, St. Louis, MO, USA). After blocking (1.5 hrs, RT in blocking solution: 3% defatted milk in 0.2% Tween 20; 20 mM Tris-HCl pH 7.5; 500 mM NaCl), membranes were incubated O/N at 4 °C with the polyclonal anti-acetyl histone H3 (lys14) antibody (07-353, Merck Millipore, Temecula, CA, USA; final concentration 0.7 μg/mL). The day after, membranes were washed 3 × 5 min in blocking buffer, then incubated 2hrs with the secondary antibody anti-rabbit IgG peroxidase (Dako, Agilent Technologies, Santa Clara, CA, USA, final concentration 0.0625 μg/mL) in blocking solution. The signal was revealed by chemiluminescence (ECL-Plus Western blotting Detection Reagents, GE-Healthcare Bio-Science, Italy) and visualized on X-ray films (BioMax Light, Kodak Rochester, NY, USA). The results were normalized *vs*. the actin signal, visualized incubating the same membrane used for revealing the H3K14Ac with the anti-actin antibody A2066 (sigma- Chemical, St. Louis, MO, USA; final concentration 0.07 μg/mL, MW 42KDa). Bands intensity was quantified by the Scion Image software (GE Healthcare Europe GmbH, France).

### ChIP-Seq analysis

The 07-353 anti-H3K13Ac antibody used for Western blot analysis was also used to perform chromatin immunoprecipitation, followed by DNA sequencing (ChIP-Seq – full result available on GEO repository # GSE109145). Chromatin immunoprecipitation (ChIP) was performed following the Magna ChIPTM G Tissue Kit (#17-20000, Merck Millipore, Temecula, CA, USA) procedure and applying the same Ab used in Western blot. Cll tissue (60 mg) was homogenized, DNA sheared (average size of 100–400 bp, by Sonopuls HD 3100, Bandelin, Germany, sonicator. Power 50%, 15″ × 18 cycles, 10″ pause between each cycle, on ice), cross-linked with 1% formaldehyde (5′, RT), and protein-DNA complexes immune-precipitated (5 μL, 07-353 Ab, Merck Millipore, Temecula, CA, USA) by G magnetic beads on the magnetic rack (LSKMAGS08 Pure ProteomeTM Magnetic Stand, Merck Millipore, Temecula, CA, USA). Protein-DNA crosslink was reversed (proteinase K, 62 °C, 2 h; plus 95° C × 10′), and DNA stored at −20 °C until use. As suggested by the manufacturer, the efficiency and specificity of the ChIP procedure were assessed by Western blot, and Real Time PCR (RTqPCR). Samples were quantified by Quant-iTTM PicoGreen® dsDNA Kits (Thermo Fisher Scientific, Waltham, MA, USA), according to manufacturer’s instruction.

Libraries were prepared by using the NEBNext® UltraTM II DNA Library Prep Kit from Illumina® (E7645, New England BioLabs®Inc, MA, USA), following the manufacturer’s instructions starting from 10 ng of fragmented DNA. After end repair and adaptor ligation, adaptor-ligated DNA clean-up (without size-selection, Agencourt AMPure XP magnetic beads, Beckman Coulter Life Sciences, CA, USA), library enrichment (98°C × 30 sec; 98°C × 10 secplus 65°C × 75 min × 10 cycles; 65°C × 5 min, in a Bio-Rad thermal cycler, Bio-Rad, Richmond, CA, USA), and PCR clean up (Agencourt AMPure XP magnetic beads, Beckman Coulter Life Sciences, CA, USA), the libraries were quantified using the PicoGreen fluorescent dye, as reported above, and stored at −20 °C. Before sequencing, libraries were denatured and diluted to a final concentration of 15 pM with 10% PhiX (Illumina, New England BioLabs®Inc, MA, USA) control. Paired-end sequencing was performed using the MiSeq reagent kit v3 2 × 150 in the Illumina® MiSeq® system (Illumina, San Diego, CA, USA). A total of 4 P9 jj Cll (2 runs) and 3 P9 control Cll (1 run) were used. Reads were mapped to the Rattus norvegicus (rn4) genome using bowtie2^46^. Duplicate reads were filtered. The quality of the sequences was evaluated using fastQC (http://www.bioinformatics.babraham.ac.uk/projects/fastqc/). Peaks were called using MACS2^47^ and annotated using HOMER software^48^. Functional enrichment study was determined using GeneCodis (http://genecodis.cnb.csic.es/, hypergeometric test, FDR corrected)^16–18^.

### Histology and morphometric analysis

Immediately after animals sacrifice, the brain was removed from the skull and fixed in 4% formalin buffered solution (4% formaldehyde 37%, 33 nM NaH_2_PO_4_, 46 mM Na_2_HPO_4_), then embedded in paraffin. Sagittal sections of the brain (3–5 μm) were obtained by a microtome (Microm-hm 340 e- BioOptica, Milan, Italy), affixed on the glass slides and dried at 60 °C for 1 hour. Hematoxylin and eosin stain (H&E) was performed by a Leica ST5020 Multistainer (Leica Microsystem, Milan, Italy). Cresyl violet (Nissl) staining was performed manually on hydrated sections (xylol 3 × 5 min; 100% ethanol 2 × 2 min; 95% ethanol 2 × 2 min; 80% ethanol 1 × 2 min; 70% ethanol 1 × 2 min; H_2_O 2 × 5 min) by incubating the slices for 1 hr in cresyl violet solution (0.1% cresyl violet powder, 10 drops glacial acetic acid in H_2_OmQ). After washing (twice H_2_OmQ), differentiation (75% ethanol, 95% ethanol plus 5% chloroform, 3 drops glacial acetic acid) and dehydration (100% ethanol 2 × 5 min; xylol 2 × 5 min), slices were mounted (Eukitt 03989, SIGMA Aldrich). Pictures were collected by a D-Sight plus image digital microscope & scanner (Menarini Diagnostics, Firenze, Italy). Histology was read by 3 independent pathologists, blinded to experimental design.

### RTqPCR on selected genes

RTqPCR was performed as previously described^26,43^. Total RNA extraction (Eurogold RNA Pure reagent, Euroclone, Milan, Italy) and retro-transcription (1 μg RNA, High Capacity cDNA Reverse Transcription Kit, Applied Biosystems, Monza, Italy) were performed following the manufacturer instruction in a thermal cycler (Gene Amp PCR System 2400, Perkin-Elmer, Boston, MA, USA) at 25 °C for 5 min, 37 °C for 120 min, and 85 °C for 5 min. The final cDNA was stored at 20 °C until use. Primers were designed using the Beacon designer 8.1 software (Premier Biosoft International, Palo Alto, CA, USA) on rat sequences available in GenBank (Table 2). RtqPCR was performed in an iCycler iQ thermocycler (Bio-Rad Laboratories, Hercules, CA, USA) in presence of 25 ng of cDNA, sense and antisense gene-specific primers (250 nM each), in SSoAdvance SYBER green supermix (Bio-Rad Laboratories, Hercules, CA, USA). Amplification protocol was 95 °C × 3 min, 40 cycle of 95° C × 20 sec; 60 °C × 20 sec and 72 °C × 30 sec, followed by 72 °C × 5 min. Melting curve analysis was performed to assess product specificity. The relative quantification was made using the iCycler iQ Software, version 3.1 (Bio-Rad Laboratories, Hercules, CA, USA) by the Pfaffl modification of the ΔΔCT equation, taking into account the efficiencies of the individual genes^49,50^. The results were normalized to the housekeeping genes and the levels of mRNA were expressed relative to a reference sample^50,51^.

**Table 2:** Primers specification.

Gene	Accession number	Forward	Revers	Efficiency	Amplicon length (bp)
*Agrn*	NM_175754	TACCTGTCCACTTGTATT	TTCTCATCCAATAACACATT	98.5	87
*Arhgap4*	NM_144740	CTTGTGAGCCATCTACTATC	GTTGAGGAAGGTGAAGAG	88	75
*Anxa2*	NM_019905	CTACTGTCCACGAAATCCTG	AAGTTGGTGTAGGGTTTGAC	99.8	94
*Casp6*	NM_031775	ACAGATGGCTTCTACAGA	AGTTCCTCTCCTCTTGTG	102.2	78
*Chmp1a*	NM_001083313	ATCAACTTACAGGTTAGG	TACTTACGACAACATTCTA	98.2	122
*Col4a3*	NM_001135759	TCACCACAATGCCATTCTTA	CGACAGCCAGTATGAATAGT	94.5	83
*Icam1*	NM_012967	ACCTACATACATTCCTACC	ATGAGACTCCATTGTTGA	96.3	91
*Mag*	NM_017190	ACCATCCAACCTTCTGTATC	CTGATTCCGCTCCAAGTG	96.2	90
*Ptk2b*	NM_017318	TGTCTACACGAACCATAA	GAACTTCTCCTTGTTGTC	93.1	88
*Tubb2b*	NM_001013886	CAGTTGGAAGAAGGAGAA	AGTGTTACATTGATGTTATCG	107.5	111
*Il6*	NM_012589.1	GCCCACCAGGAACGAAAGTC	TCCTCTGTGAAGTCTCCTCTCC	107.7	161
*Hprt*	NM_012583.2	AGACTGAAGAGCTACTGTAATGAC	GGCTGTACTGCTTGACCAAG	94.9	163

### Statistics

The statistical analysis was performed by GraphPad InStat for Windows (GraphPad Software, Inc, La Jolla, CA, USA). The ANOVA test, followed by Tukey-Kramer multiple comparison tests, was used to analise TSB, cBf, and Cll weight during the development. The unpaired two-tailed Student’s t-test, based on unequal variance, was applied to evaluate the difference between jj and controls at the same age (Western blot, RTqPCR). All data are expressed as mean ± S.D. of multiple biological repetition. A p-value lower than 0.05 was considered statistically significant.

## Data Availability

ChIP-Seq – full result available on GEO repository # GSE109145.
